# Insight Into the Effects of Clinical Repetitive Transcranial Magnetic Stimulation on the Brain From Positron Emission Tomography and Magnetic Resonance Imaging Studies: A Narrative Review

**DOI:** 10.3389/fnins.2022.787403

**Published:** 2022-02-21

**Authors:** Lucero Aceves-Serrano, Jason L. Neva, Doris J. Doudet

**Affiliations:** ^1^Department of Medicine/Neurology, University of British Columbia, Vancouver, BC, Canada; ^2^École de Kinésiologie et des Sciences de l’Activité Physique, Faculté de Médecine, Université de Montréal, Montréal, QC, Canada; ^3^Centre de Recherche de l’Institut Universitaire de Gériatrie de Montréal, Montréal, QC, Canada

**Keywords:** rTMS (repetitive transcranial magnetic stimulation), PET, resting state connectivity (rsfMRI), MRS (magnetic resonance spectroscopy), diffusion tensor imaging (DTI), animal models

## Abstract

Repetitive transcranial magnetic stimulation (rTMS) has been proposed as a therapeutic tool to alleviate symptoms for neurological and psychiatric diseases such as chronic pain, stroke, Parkinson’s disease, major depressive disorder, and others. Although the therapeutic potential of rTMS has been widely explored, the neurological basis of its effects is still not fully understood. Fortunately, the continuous development of imaging techniques has advanced our understanding of rTMS neurobiological underpinnings on the healthy and diseased brain. The objective of the current work is to summarize relevant findings from positron emission tomography (PET) and magnetic resonance imaging (MRI) techniques evaluating rTMS effects. We included studies that investigated the modulation of neurotransmission (evaluated with PET and magnetic resonance spectroscopy), brain activity (evaluated with PET), resting-state connectivity (evaluated with resting-state functional MRI), and microstructure (diffusion tensor imaging). Overall, results from imaging studies suggest that the effects of rTMS are complex and involve multiple neurotransmission systems, regions, and networks. The effects of stimulation seem to not only be dependent in the frequency used, but also in the participants characteristics such as disease progression. In patient populations, pre-stimulation evaluation was reported to predict responsiveness to stimulation, while post-stimulation neuroimaging measurements showed to be correlated with symptomatic improvement. These studies demonstrate the complexity of rTMS effects and highlight the relevance of imaging techniques.

## Introduction

Transcranial magnetic stimulation (TMS) is a brain stimulation technique that modulates activity non-invasively. TMS accomplishes this via the induction of electrical currents through rapidly changing magnetic field pulses. When TMS is applied in repetitive trains of stimulation (i.e., repetitive TMS, rTMS), its effects on cortical excitability can outlast the period of stimulation (for review, see Fitzgerald et al. (2006), Nordmann et al. (2015)). In humans, rTMS has been shown to induce plasticity reflected as changes in corticospinal excitability. Changes in excitability are often measured using motor evoked potential (MEP) amplitude, elicited with single-pulse stimulation to the motor cortex (Pell et al., 2011; Nojima and Iramina, 2018). rTMS can be used to either increase (excitatory rTMS) or decrease (inhibitory rTMS) cortical excitability depending on the paradigm of repetitive stimulation used (Speer et al., 2000; Lang et al., 2006). The ability of rTMS to modulate cortical excitability beyond the period of repetitive stimulation makes it an appealing therapeutic tool for neuropsychiatric disorders where plasticity induction proves helpful (for review, see Lefaucheur et al. (2020)).

Repetitive transcranial magnetic stimulation is currently approved by regulatory agencies as a therapeutic tool for drug-resistant depression (O’Reardon et al., 2007) and obsessive-compulsive disorder (Carmi et al., 2018). Studies have also proposed rTMS delivery as a potential therapeutic tool in the treatment of multiple disorders such as drug addiction (Diana et al., 2017), gambling disorder (Moccia et al., 2017), neuropathic pain (Lefaucheur, 2006a; Andre-Obadia et al., 2018), stroke (Corti et al., 2012; Neva et al., 2020), and dystonia (Siebner et al., 2003; Kimberley et al., 2015). However, the mechanisms through which rTMS elicits its therapeutic effects are still to be elucidated. For example, while the approved excitatory rTMS delivery on the left dorsolateral prefrontal cortex (DLPFC) is effective in alleviating depression symptoms (Perera et al., 2016), recent studies suggest that inhibitory rTMS of the right DLPFC may be as effective (Schaffer et al., 2021).

To fully exploit the potential of rTMS in clinical settings, it is necessary to understand rTMS-induced neurochemical and functional changes directly in the human brain. The emergence and constant development of brain imaging techniques have facilitated the study of rTMS effects. Positron emission tomography (PET) and magnetic resonance imaging (MRI) techniques such as diffusion tensor imaging (DTI) and magnetic resonance spectroscopy (MRS) can be used to evaluate functional, microstructural, and neurochemical changes following stimulation.

For example, the availability of multiple PET radiotracers makes PET imaging a relevant tool in studying the brain’s physiology. This neuroimaging tool can be used to measure the modulation of specific neurotransmitters and receptor systems, taking advantage of the development of a variety of radioligands for receptors, transporters, enzymatic activity, and neurotransmission processes. MRS can also be used to evaluate changes in neurotransmitter concentrations such as gamma-aminobutyric acid (GABA) and glutamate, the major inhibitory and excitatory neurotransmitters in the brain, respectively. Further, MRI and PET techniques can be used to evaluate neural activity modulation. Specifically, 2-deoxy-2-(18F) fluoro-D-glucose (FDG), a PET tracer and glucose analog, has been used to evaluate rTMS induced changes in neuronal activity. [^18^F]FDG uptake reflects glucose metabolism in neural tissue and, given that glucose metabolism increases during neuronal activation (Raichle and Mintun, 2006). Similarly, PET O^15^H_2_O measures neural activation indirectly by recording regional cerebral blood flow (rCBF) changes, which are coupled to brain activity.

Functional magnetic resonance imaging (fMRI) measurements of blood-oxygen-level-dependent (BOLD), a measurement modulated by neural activity (Raichle and Mintun, 2006). Fluctuations in the BOLD signal across the brain can be used to evaluate functional connectivity, a method that identifies correlation patterns between brain regions (Büchel and Friston, 1997; Greicius et al., 2004).

These fMRI images can be acquired at rest (rsfMRI) or during a task. During a task, fMRI is used to record task-derived changes in brain activity, while at rest fMRI is used to detected regions in which patterns of spontaneous BOLD fluctuation correlate when participants are at rest. Both methods have proven helpful in their way as they can evaluate different processes or mechanisms of rTMS. Task-based studies can evaluate activation pattern discrepancies between healthy and patient populations, as well as inquire how these patterns are affected by rTMS delivery. Resting state fMRI can be used to record the effect of rTMS in functional connectivity and avoid the measurement of task-derived modulation. In addition, MR techniques such as DTI can be used to evaluate rTMS induced changes in white matter microstructure and structural connectivity.

Given the broad literature of neuroimaging studies investigating the effects of rTMS, in this narrative review, we present a summary of some of the relevant findings from PET and MRI studies in humans and large animal models. The literature search was carried out with PubMed using major terms such as “rTMS” or “repetitive transcranial magnetic stimulation” in combination with PET, MRS, resting-state connectivity, or DTI.

We have organized the current work in distinct sections, starting with an introduction to rTMS protocols and some of the disorders relevant to the current work, along with a small section on rTMS and *in vivo* imaging in animal models. We then present literature in human participants evaluating *in vivo* neurotransmission systems (using PET and MRS), activity modulation (using PET tracers for blood flow and glucose metabolism), functional connectivity (using rsfMRI), and microstructure (using DTI).

## Repetitive Transcranial Magnetic Stimulation Overview

The diversity of rTMS protocols enabled the study of its applicability and effectiveness in brain disorders. Several rTMS parameters can be modified depending on the target population, including the stimulated area, stimulus frequency and patterns, stimulus intensity, duration of the application period, and the total number of stimuli. Conventionally, low-frequency rTMS (LF-rTMS, ≤1 Hz) is consider to decrease cortical excitability while high-frequency rTMS (HF-rTMS, ≥5 Hz) is consider to increase it (Houdayer et al., 2008). In addition to the conventional HF- and LF-rTMS, several patterned protocols with more complex timings exist, such as theta-burst stimulation (TBS) and quadripulse stimulation (QPS), which can increase and decrease cortical excitability depending on the interval between patterns of stimuli (i.e., bursts).

Theta-burst stimulation is delivered in bursts of 3 stimuli at 50 Hz, every 2 s, and can be delivered continuously (cTBS) or intermittently (iTBS), inducing a decrease or increase in cortical excitability, respectively. As its name indicates, cTBS involves the application of continuous TBS pulses for 20 to 40 s (300 to 600 stimuli, respectively), whereas, in iTBS, the TBS pattern is applied in a 10-s intertrain interval, with 2 s of stimulation and 8 s of no stimulation, for a total of 190 s (Huang et al., 2005).

Quadripulse stimulation consists of bursts of four monophasic magnetic pulses at 0.5-ms intervals for 30 min to 360 bursts. Like TBS, QPS can induce an increase or decrease in cortical excitability. QPS delivered with a short inter-pulse interval (smaller than 15 ms) increases excitability, while QPS delivered with larger intervals (between 15 and 100 ms) decreases it (Hamada et al., 2008).

In addition to different frequencies and patterns, rTMS effects can also be altered by the coil used. There are multiple coils designs and sizes available for rTMS delivery. Using a different coil affects the size, shape, and depth of the induced electrical field in the brain (Deng et al., 2013). Generally, the depth of stimulation from a given coil can be estimated by dividing the coil diameter in half. For example, a 7 cm diameter coil induced a stimulation depth of 4.5 cm depth starting from the coil, which must penetrate the plastic casing of the coil, the skull, and cerebrospinal fluid before reaching brain tissue. Current human studies mainly use a figure-eight coil, which reaches cortical stimulation depth ranging from 1 to 1.9 cm in the cortex (Deng et al., 2013). Circular coils are often used in clinical populations in specific cases where the depth of stimulation might be more critical than stimulation focality, which tends to be compromised in circular coils compared to figure-eight coils. Deeper brain regions can also be targeted using specialized coils, such as an H-coil, enabling the targeting of deeper structures (>2.4 cm in brain matter) bilaterally (Deng et al., 2013).

Parameters such as the stimulus intensity can and is often individualized according to the resting or active motor threshold (RMT and AMT, respectively). Motor thresholds are measured by delivering single-pulse TMS over the motor cortex and assessing the MEPs via electromyographical (EMG) measurement in the targeted muscle. RMT is measured while the muscle is at rest, and it is defined as the minimal intensity of a single pulse of TMS required to elicit an MEP with a peak-to-peak amplitude ≥50 μV in at least 5 out of 10 consecutive trials (Boroojerdi et al., 2001). AMT is obtained the same way but during an isometric contraction of 5–20% of the maximal voluntary contraction, and where the peak-to-peak MEP amplitude needs to be ≥200 μV in at least 5 out of 10 consecutive trials (Berardelli et al., 2008; Boyd et al., 2014; Auriat et al., 2015).

### Main Therapeutic Applications of Repetitive Transcranial Magnetic Stimulation

#### Depressive Disorders

Mood disorders are chronic disorders characterized by the occurrence of depressed mood along with a loss of interest in activities that were once enjoyable. The pathophysiology of mood disorders is still not fully understood but studies have shown patients present hypoactivity in the left and hyperactivity in the right frontal cortex. This led to the application of facilitatory rTMS over the left or inhibitory rTMS over the right frontal cortex (Fitzgerald et al., 2009; George et al., 2010). Similarly, rTMS studies of other neuropsychiatric and mood disorders have been based on the induction of cortical excitability decrease or increase (see Lefaucheur et al. (2014b)).

#### Parkinson’s Disease

Parkinson’s disease is characterized by a progressive loss of dopaminergic neurons in the substantial nigra, disrupting the basal ganglia-thalamo-cortical loops and regions, including the primary motor cortex (Burciu and Vaillancourt, 2018). Parkinson’s patients manifest various motor symptoms such as bradykinesia, tremors, rigidity, and gait difficulties (Moustafa et al., 2016). While Parkinson’s motor symptoms are the most clinically evident, non-motor signs such as cognitive dysfunction and depression are also prominent in Parkinson’s patients (Helmich et al., 2006). Similar to mood disorders patients, studies have indicated that Parkinson’s disease is accompanied by motor cortices dysfunction (Lefaucheur, 2005), thus making them a sound targets for rTMS delivery.

#### Stroke

Following a stroke, changes in brain activity in both hemispheres are observed, these changes can be advantageous, but they can also lead to maladaptive recovery (Ward et al., 2003; Altman et al., 2019). Studies have suggested a shift in sensorimotor cortical excitability in both hemispheres (Dijkhuizen et al., 2003), where excitability decreases in the affected region and increases in the unaffected area (Huynh et al., 2016). Thus, LF-rTMS delivery over the unaffected hemisphere has been used to improve motor performance by rebalancing the excitability of the hemispheres (Lefaucheur, 2006b). While fewer studies have delivered HF-rTMS ipsitralesionally, beneficial results using this method have also been reported (see Lefaucheur et al. (2014a)).

#### Chronic Pain

Chronic pain research suggested that the disorder might be associated with maladaptive plastic changes in the central and peripheral nervous system (Fregni et al., 2007). Several areas, including limbic, thalamic, and cortical regions such as the sensorimotor, insular, parietal, and prefrontal cortex, have been suggested as regions contributing to a state of over-reactivity (Goossens et al., 2018; Ong et al., 2019). Researchers have explored the potential of rTMS when applied over the motor cortex under the assumption that pain perception could be modulated by indirect effects carried via neural networks that ultimately target pain-modulating areas such as the thalamic and limbic regions (Lefaucheur et al., 2020).

#### Addiction

Addiction is a complex disorder involving several neural pathways; the main implicated pathways are those from the meso-cortico-limbic system (Koob and Volkow, 2010). This condition is characterized by the disruption and dysregulation of dopaminergic activity induced by exposure to addictive substances (Volkow et al., 2007). Thus, delivery of rTMS in patients with addiction is thought to be able to modulate connectivity between regions of the mesolimbic system and alleviate symptoms by altering activity in relevant cortices, such as the hypoactive frontal cortex (Hu et al., 2019).

## Imaging Methods in Repetitive Transcranial Magnetic Stimulation Studies

### Positron Emission Tomography Studies of Neurotransmission

Positron emission tomography imaging relies on detecting and localization of a radionuclide used to label a molecule (e.g., receptor/transporter agonists or antagonists, enzymatic precursors) to target a specific neurotransmitter system. Various radiotracers can be used to monitor specific proteins and neurochemicals processes in the living brain non-invasively. PET-rTMS studies have been heavily focused on evaluating stimulation effects on endogenous dopamine. The majority of such studies use [^11^C]raclopride, a D_2/3_ receptor antagonist (Laruelle, 2000), as a surrogate marker for dopamine level quantification in the striatum. Evaluation of extrastriatal dopamine release can also be assessed using higher-affinity receptor radioligands like [^18^F]fallypride (Hernaus and Mehta, 2016) and [^11^C]PHNO (Wilson et al., 2005).

Positron emission tomography imaging has also been used to evaluate rTMS-induced perturbations in other neurotransmitters such as serotonin and endogenous opioids. Serotonin imaging is enabled by tracer such as [^11^C]αMtrp synthetic analog of the essential amino acid precursor to 5HT, L-tryptophan (Trp), which provides a proxy measure for serotonin synthesis capacity *in vivo*. Imaging of the endogenous opioid system can be monitored using [^11^C]carfentanil as a high-affinity μ opioid receptor antagonist.

While understanding rTMS-driven neurotransmitters release is an essential tool to understand the short- and long-term mechanisms of action of the non-invasive stimulation therapies, other tracers also permit measuring and quantifying other processes, such as inflammation or synaptic density.

### Magnetic Resonance Spectroscopy Imaging

Magnetic resonance spectroscopy allows to investigate concentrations of metabolites *in vivo* non-invasively and can thus can be used as a piece of surrogate information to infer neurotransmitter and neurochemical changes. Usually, MRS is acquired from a volume of interest containing several voxels at a time (1 cm^3^ is often considered adequate size). Its acquired spectral components from *N-*acetyl aspartate (NAA), total NAA (tNAA), a combination of NAA and NAA glutamate (NAAG), creatine (Cr), phosphocreatine (PCr), choline, myoinositol, and the combined signal of glutamate and glutamine (Glx).

Under specific acquisition and analysis conditions, it is also possible to record spectral components of glutamate and glutamine separately and gamma-aminobutyric acid (GABA). MRS measurements of GABA and glutamate concentration can be used to monitor inhibitory and excitatory neurotransmission, while the other metabolites present in the MRS spectra have been used as markers of other neurochemical processes.

Quantification of GABA is technically challenging due to its low concentration (Govindaraju et al., 2000) and spectral overlap with other metabolites that are more abundant (Puts and Edden, 2012). One possible method to separate the GABA spectra is to use scanners with higher field strength, such as 7Tesla (Gröhn et al., 2019). Alternatively, spectral editing and two-dimensional MRS methods can be used when higher field scanners are unavailable. Spectral editing methods such as MEGA-PRESS are used to reduce the information in the spectrum (Buonocore and Maddock, 2015), while two-dimensional MRS methods can spread the overlapping signals (Ke et al., 2000).

Glutamate (Glu) spectra overlap with glutamine (Gln) spectra, making it simpler to report information from both spectra together as Glx. Modifying echo times to shorter ones can improve the detection of Glu (Wilson et al., 2019), as done with sequences such as SPECIAL and sLASER (Öz et al., 2021).

### *In vivo* Imaging of Neural Activity and Functional Connectivity

Positron emission tomography evaluation of neural activity is enabled by measuring alterations in [^18^F]FDG, a glucose analog, and flow and oxygen metabolism using ^15^OH_2_O and ^15^O radiotracers. As neural activity changes, changes in glucose consumption and metabolism are reflected, leading to an increase or decrease in the uptake and trapping of [^18^F]FDG. PET- ^15^OH_2_O imaging is used to measure regional cerebral blood flow (rCBF), coupled with neural activity, unless there are severe pathological conditions where uncoupling may occur (Raichle and Mintun, 2006).

Another helpful imaging tool to evaluate neural activity is fMRI. Similar to PET- O^15^H_2_O, this technique can monitor changes in blood flow through the measurement of the BOLD signal and change in oxygenation as an indirect measure of neural activity. When acquired at rest, fMRI can identify the correlation of spontaneous fluctuations in brain activity among spatially distributed brain regions; this analysis is defined as resting-state functional connectivity (rsFC) (Biswal et al., 2010; Smitha et al., 2017). In rTMS studies, rsFC evaluation is useful as it allows for the assessment of rTMS effects without accounting for connectivity modulation concomitant to a task or state.

Regions that show statistical dependence in BOLD signal fluctuation are known as resting-state networks (Smith et al., 2009; Doucet et al., 2011; Hamada et al., 2013; Smitha et al., 2017). These networks can be identified using several analysis techniques, seed-based analysis being the first method adopted in early work by Biswal et al. (1995). Such analysis finds the linear correlation of a pre-selected region (a seed region) with all the other voxels in the brain. While this is advantageous due to its simplicity and interpretability, its results are heavily influenced by the seed region selected.

Later techniques have allowed hypothesis-free analysis of rs-fMRI data; one of such techniques is independent component analysis (ICA) (Beckmann et al., 2005; Damoiseaux et al., 2006; Bi et al., 2018) which allows for the distinction of networks through decomposition of the signal from all brain voxels to temporal and spatial independent components. In addition, techniques such as k-means clustering (Thomas Yeo et al., 2011; Zhang and Li, 2012; Drysdale et al., 2017), graph theory (Lord et al., 2012; Khazaee et al., 2015), and others (for review see Khosla et al. (2019)), have expanded the available toolbox of hypothesis-free rs-fMRI analysis methods.

### *In vivo* Imaging of Structural Integrity Changes With Diffusion Tensor Imaging

Diffusion tensor imaging can evaluate structural integrity and structural changes in white matter. It allows the acquisition of diffusion water maps in the brain and provides quantitative information through diffusion coefficients (Alexander et al., 2007; Westlye et al., 2010; Hawco et al., 2018). Diffusion maps provide microstructural information that allows the detection of changes in white matter and can also be used to evaluate alterations in neuroanatomy due to aging (Poudel et al., 2015; Lebel and Deoni, 2018), disease (Melzer et al., 2013; Poudel et al., 2015; Visser et al., 2019) or interventions (Coenen et al., 2014; Pi et al., 2019).

Fractional anisotropy (FA) measurements described the directionality of random water diffusion (Pierpaoli and Basser, 1996). FA values range from 0 (isotropic) to 1 (anisotropic), where values closer to 0 represent water molecules that diffuse freely in any direction or are equally restricted in all directions (e.g., cerebral fluid) and values closer to 1 represent water diffusion occurring in a single direction (e.g., when contained in fiber bundles). Regarding white matter FA estimates, higher values are observed in heavily myelinated tracks, given that the myelin restricts the diffusion of water molecules to the direction along the fiber tracks (Beaulieu, 2002). Another commonly used DTI measurement is mean diffusivity (MD) representing the average mobility of water molecules where higher values of MD indicate a more isotropic movement.

Diffusion tensor imaging can provide information on the local white matter bundle orientation, which can be used to reconstruct white matter connections (Lin et al., 2011) and structural connectivity (Chiang and Haneef, 2014). DTI tractography analyzed using graph theory methods can evaluate fiber count as a measurement connectivity strength (Caeyenberghs et al., 2012). Specifically, metrics to evaluate global or local connectivity such as characteristic path length, global efficiency, strength, and modularity can be obtained (Kocevar et al., 2016; Yu et al., 2018). Path length is a measurement of the information integration processing, an increase in this measurement represents a bigger number of steps for brain regions to communicate with each other. Global efficiency is a proportional inverse of path length, and it provides information about the network’s parallel information processing efficiency. Finally, modularity and strength are measurements that evaluate the network segregation and the weight of the connectivity across the regions of a network (Girvan and Newman, 2002; Baum et al., 2017).

## Imaging Studies of Repetitive Transcranial Magnetic Stimulation Effects in Animal Models

Even though the current review focused on human imaging studies, it is important to acknowledge the value of animal work in unveiling the mechanisms of rTMS. The use of animal models provides a unique opportunity compared to human studies; it allows the parallel evaluation of behavior and non-invasive *in vivo* imaging and permits confirmation of the *in vivo* findings and monitoring of molecular and cellular changes using invasive techniques, including postmortem evaluations. Furthermore, rTMS studies in animal subjects decrease population variability by controlling for subject characteristics such as age, sex, disease stage, and genetic background. Animal models can also evaluate rTMS safety as the effects of higher frequency, a larger number of pulses and supra-threshold intensities can be measured on the cellular, molecular and inflammatory responses.

Non-human primates are a precious animal model in the study of the brain, and they are an essential tool for understanding the neural mechanisms involved in non-invasive brain stimulation techniques. Specifically, in evaluating rTMS effects, non-human primates provide several advantages compared to other animal models. Their large brains and comparable neuroanatomy to humans facilitate the translation of the methods with only slight adjustments and even permit the use of standard coils as well as facilitate the use and comparison of neuroimaging data from PET and MR imaging (Ohnishi et al., 2004; Salinas et al., 2013, 2015). Non-human primates can be trained to sit in a monkey chair (McMillan et al., 2014) and receive rTMS daily while awake (Aceves-Serrano et al., 2019a,b). The variety of available primate models of diseases such as Parkinson’s disease, epilepsy, and stroke can be used to provide insights into the effects of rTMS in these different disorders (Bezard et al., 1997; Szabó et al., 2005; Kadono et al., 2021). Finally, healthy non-human primates could help to evaluate the impact of a clinical course of rTMS delivery and allow for comparison between the effects of rTMS in the healthy and non-healthy brain.

In monkeys, a [^11^C]raclopride studies of acute stimulation reported an increase in striatal dopamine release following HF-rTMS (Ohnishi et al., 2004) and a decrease after cTBS (Aceves-Serrano et al., 2019a). In addition, PET studies of glucose metabolism in healthy monkeys showed rTMS over the motor cortex induced immediate and long-lasting (8 and up to 16 days after stimulation) effects in areas anatomically of functionally connected to the targeted region (Hayashi et al., 2004), which were also observed 16 days after stimulation. In healthy baboons, Salinas et al. (2013, 2016) showed that different stimulation frequencies yield distinct effects in rCBF. Authors found both increase and decrease in rCBF after different HF rTMS frequencies (3 Hz, 5 Hz, 10 Hz, and 15 Hz), and their work suggested different frequencies might be optimal for targeting different nodes from the motor network. This study, in particular, highlights the advantage of animal imaging studies as such study design would not have been feasible in human subjects due to ethical concern of radioactivity exposure, as multiple scans per subject were acquired.

Magnetic resonance studies of rTMS effects have also been carried in animals. In a non-human primate model of post-stroke pain, fMRI evaluation demonstrated HF-rTMS over the ipsilesional motor cortex induced pain relief and changes in connectivity between the thalamus and the amygdala (Kadono et al., 2021). While, in rats, a resting-state fMRI study showed different rTMS frequencies and patterns (1 Hz, 10 Hz, continuous theta-burst stimulation (cTBS) or biomimetic high-frequency stimulation) delivered over the right hemisphere modulated connectivity in the somatosensory cortex, the motor cortex, and the hippocampus (Seewoo et al., 2018). Like what was reported in monkeys and humans, effects in these regions were frequency-dependent.

Taken together, these studies show the feasibility of rTMS studies in animal models. They illustrate the advantages that animal studies provide, such as the acquisition of multiple imaging studies per subject and the evaluation of the effects of multiple rTMS parameters in the same animal. In addition, rTMS imaging studies allow direct comparison between the results from human and extensive animal studies, as similar acquisition and analysis parameters are typically implemented. Importantly, in the case of fMRI evaluation, the identification of resting-state networks in monkeys and rodents (Vincent et al., 2007; Hutchison and Everling, 2012; Lu et al., 2012) simplifies the translation of resting-state fMRI results from animal to humans.

## Positron Emission Tomography Evaluation of Repetitive Transcranial Magnetic Stimulation Effects in Neurotransmission

### Dopamine

#### Healthy Participants

An early PET study in healthy participants by Strafella et al. (2001) detected a striatal decrease of [^11^C]raclopride binding immediately after HF-rTMS over the left prefrontal cortex (PFC), indicating an increase in dopamine release in the left caudate nucleus. The same research group later showed a similar increase in the putamen after HF-rTMS over the left motor cortex (Strafella et al., 2003).

Dopamine imaging studies have also reported a decrease in dopamine release following rTMS administration. One study by Ko et al. (2008) looked at the effects of inhibitory cTBS over the left dorsolateral DLPFC before a planning and set-shifting task. Authors found that cTBS, an inhibitory form of rTMS, impaired performance and decreased dopamine release in the bilateral caudate and the putamen. Given the study design, it is unclear whether this bilateral effect resulted from the higher frequency (50 Hz), the task at hand, or a combination of the two.

A later study also reported a bilateral dopamine decrease in the striatum of healthy subjects following inhibitory rTMS (Malik et al., 2018). LF-rTMS was delivered to the bilateral insular cortex, which most likely mediated the bilateral response. They reported an increase in [^11^C] PHNO, a D_2_ receptor agonist, in parts of the striatum, suggesting a decrease in dopamine release. Importantly, even though Ko and Malik’s studies used different rTMS parameters (different frequency, coil, number of pulses, and target region), they both showed a decrease in striatal dopamine. These findings suggest that conventionally inhibitory forms of rTMS (LF-rTMS and cTBS) decrease dopamine release in the striatum and accord with a hypothesized decrease in cortical excitability. Notably, the changes in striatal dopamine reported in these studies follow the distribution of corticostriatal projections reported in anatomical and physiological studies (Alexander et al., 1986), suggesting dopamine release might have been mediated by modulation of the corticostriatal projections.

Delivery of HF-rTMS has also been shown to modulate extrastriatal dopamine in healthy participants. In work by Cho and Strafella (2009), the authors found dopamine increases in the anterior cingulate cortex and the orbitofrontal cortex following HF-rTMS over the left DLPFC likely facilitated by the dense anatomical connections between the DLPFC and the cingulate cortex (Petrides and Pandya, 2002). These results showed rTMS ability to indirectly increase or decrease dopamine release in multiple brain regions of healthy controls.

#### Clinical Populations

Further studies confirmed tonic striatal dopamine release induced by rTMS in patient populations. In a study of unilateral parkinsonian patients, researchers showed increased dopamine release in the bilateral putamen after HF-rTMS delivery over the symptomatic M1 (i.e., hemisphere contralateral to the clinically affected hemibody) (Strafella et al., 2005). Following work Kim et al. (2008) confirmed these findings and reported improvement of parkinsonian motor symptoms and an increase in striatal dopamine release. While the dopamine-rTMS studies previously listed have assessed the effects on a single session of rTMS, studies in participants with psychiatric conditions have been carried a clinical therapeutic course of stimulation. In subjects with depression, studies using [^11^C]raclopride and L-[b ^11^C]DOPA failed to detect changes in striatal dopamine release and dopamine synthesis (Kuroda et al., 2006, 2010) the day after ten daily HF-rTMS sessions of the left DLPFC. RTMS parameters such as frequency (10 Hz) and intensity (100% RMT) were similar to acute studies where dopaminergic changes were detected. Thus, the discrepancy might have resulted from the time between the rTMS application and the scan acquisition.

Using a similar rTMS parading, Pogarell et al. (2006, 2007) evaluated both the immediate and chronic effects of rTMS. Instead of PET imaging, the authors used single photon-emission computed tomography (SPECT) and [^123^I] IBZM, a D_2/3_ receptor antagonist. Using a bolus plus constant infusion scanning paradigm allowed authors to measure the tracer and the changes in dopamine release over hours, in this case before and after an rTMS session. In their study design, Pogarell et al. (2006, 2007) delivered rTMS daily on the left DLPFC of patients with depression for 3 weeks with an IBZM scan on day one and an IBZM scan on the last day. In each scan day, two acquisitions were performed: one before and one after the rTMS session. Tracer binding in the bilateral striatum before the first and before the last stimulation session (i.e., 24 h after the previous rTMS session but before the last session) was comparable, replicating Kuroda’s work finding (Kuroda et al., 2006, 2010). In contrast, tracer binding after the first and after the last rTMS was increased compared to their respective baseline assessment, suggesting that in both cases, after the first and after the last rTMS session, an immediate increased dopamine release in response to the stimulation. Changes in dopamine release were also accompanied by significant symptomatic improvement.

Three observations can be drawn from these results: first, the findings of the therapeutical improvements from HF-rTMS over the left DLPFC in depression are consistent. Second, measurable rTMS modulation of dopamine is transient and is not detectable within hours following the end of HF-rTMS stimulation, in line with results from a dialysis study in rats, where rTMS induced dopamine release lasted about 140 min (Kanno et al., 2004). Third, acute dopamine modulation is similar in response to HF-rTMS when delivered for the first time and delivered after multiple daily sessions (Pogarell et al., 2006, 2007). However, these studies were carried in a small sample of patients with depression, and it is not clear whether the subjects’ neuropathology drove or at least influenced these results.

In summary, PET studies in healthy subjects and patient populations have shown that HF-rTMS delivery increases dopamine in the ipsilateral striatum (Table 1). Dopamine modulation is localized in areas that correspond to the distribution of the stimulated corticostriatal projections. In human subjects with Parkinson’s disease and mood disorders have shown bilateral changes in striatal dopamine transmission, which highlights how differences in the neurochemical and functional state (disease compared to healthy control) may influence rTMS effects on striatal dopamine release (ipsilateral vs. bilateral) (Ko and Strafella, 2012).

**TABLE 1:** SPECT and PET dopamine studies of rTMS effects in healthy controls and clinical populations.

		rTMS				
Author	Subjects	Frequency, intensity	Sessions (pulses/session)	Target region	Tracer	Results
Strafella et al., 2001	*N* = 8, HC	10 Hz, 100% RMT	1 (450)	L DLPFC	[^11^C]raclopride	↑ DA in ips caudate
Strafella et al., 2003	*N* = 6, HC	10 Hz, 90% RMT	1 (450)	L M1	[^11^C]raclopride	↑ DA in ips putamen
Strafella et al., 2005	*N* = 7, PD	10 Hz, 90% RMT	1 (450)	More affected M1 Less affected M1	[^11^C]raclopride	↑ DA in ips putamen. Greater release in less affected M1.
Kuroda et al., 2006	*N* = 9, MD	10 Hz, 100% RMT	10 (1000)	L DLPFC	[^11^C]raclopride	NS
Pogarell et al., 2006, 2007	*N* = 5, MD	10 Hz, 100% RMT	1 (3000) 15 (1500)	L DLPFC	[^123^I] IBZM	↑ DA L and R striatum
Ko et al., 2008	*N* = 10, HC during a task	50 Hz (cTBS), 80% AMT	1 (900)	L DLPFC, vertex (control)	[^11^C]raclopride	↓ DA L and R caudate and left putamen
Kim et al., 2008	*N* = 9, PD	5 Hz, 90% RMT	2 (150)	More affected M1	[^11^C]raclopride	↑ DA contralateral caudate
Cho and Strafella, 2009	*N* = 7, HC	10 Hz, 100% RMT	1 (750)	L DLPFC, R DLFPC	[^11^C]FLB 457	↑ DA L ACC and mOFC post left DLPFC rTMS
Kuroda et al., 2010	*N* = 8, MD	10 Hz, 100% RMT	10 (1000)	L DLPFC	L-[β^−11^C]DOPA	NS
Lamusuo et al., 2017	*N* = 11, HC	10 Hz, 90% RMT	1 (1000)	L DLPFC	[^11^C]raclopride	NS
Malik et al., 2018	*N* = 8, HC	10 Hz, 120% RMT 1 Hz, 120% RMT	1 (1000) 1 (1020)	Bilateral insula (H-coil)	[^11^C] PHNO	↑ DA L and R SN (1 Hz) ↑ DA L and R SMST (1 Hz)

*↑, increase; ↓, decrease; ACC, anterior cingulate cortex; bi, bilateral; DA, dopamine release; DAT, dopamine transporter; DLPFC, dorsolateral prefrontal cortex; GD, gambling disorder; HC, healthy control; ips, ipsilateral; L, left; M1, motor cortex; MD, mood disorder; mOFC, medial orbitofrontal cortex; NS, non-significant; PD, Parkinson’s disease; R, right; SMST, sensorimotor striatum; SN, substantia nigra.*

A significant limitation of some of the studies presented here is the lack of a control condition (Strafella et al., 2005; Kuroda et al., 2006, 2010; Pogarell et al., 2006, 2007; Cho and Strafella, 2009), which is specifically relevant in studies of dopamine release modulation. Such study design may make measurement susceptible to expectation, leading to dopamine release (De la Fuente-Fernández et al., 2001).

### Other Neurotransmitters

Positron emission tomography studies of rTMS effects have primarily focused on evaluating dopamine modulation, partly because of the relevance of the dopaminergic system and the availability of tracers for dopaminergic evaluation. However, a few PET studies have evaluated the effect of rTMS on serotonin and endogenous opioid release.

An early pilot study used [^11^C]-Alpha-methyl-tryptophan ([^11^C]-αMtrp), a synthetic analog of the serotonin precursor, and measured “trapping” as a proxy measurement for or serotonin synthesis capacity *in vivo*. Their results suggested that HF-rTMS delivered over the left DLPFC could modulate serotonin metabolism and trapping in parts of the limbic cortex of healthy subjects right after a single stimulation session (Sibon et al., 2007). These results are in line with reported results from SPECT imaging, where binding of [^123^I]-5-I-R91150, a 5-HT_2A_ receptor antagonist decreased following 10 sessions of rTMS delivery (Baeken et al., 2011). These changes in serotonin release correlated with symptomatic improvement, suggesting that HF-rTMS therapeutic effects might be associated with modulation of serotonin release and metabolism.

A study by Lamusuo et al. (2017) evaluated the effects of HF-rTMS in both the dopaminergic and opioid systems. While they found no dopamine release changes hours after the end of HF-rTMS, the evaluation using an agonist tracer of the μ opiate receptor ([^11^C]carfentanil) continued to show significant changes more than 5 h after the end of the stimulations.

Although there is limited literature on the effects of rTMS on neurotransmitters other than dopamine, these PET and SPECT studies highlight the complex multi-neurotransmitter effect of rTMS. The effects of rTMS on dopamine release seem to be immediate and transient, given the lack of effects after several hours (Kuroda et al., 2010; Lamusuo et al., 2017). In contrast, studies of the serotonergic system reported immediate [right after stimulation (Sibon et al., 2007)] and longer-lasting effects [a week after 10 daily sessions (Baeken et al., 2011)]. Finally, there also seems to be an effect of rTMS in the opioid system, which lasts longer than the effect in the dopaminergic system (Lamusuo et al., 2017). These studies confirm that different neurotransmission systems have a unique response to rTMS, with modulation lasting from less than a few hours to several days.

Neuropharmacological imaging is limited to a few neurotransmitters and changes in the two transmitters that may present the most interest based on their ubiquitous distribution, GABA and glutamate, are not easily measurable by PET or SPECT. Combination with other modalities, as described below, may, however, permit one to have a glimpse into their role.

## Magnetic Resonance Spectroscopy Evaluation of Repetitive Transcranial Magnetic Stimulation Effects in Neurotransmission

In this section, we present results from MRS literature focused on investigating the effects of rTMS on neurotransmission. In this review, we have not included studies that only report modulation of metabolites other than GABA, Glu, and Glx [e.g., Myo-inositol, choline, and *N*-acetyl aspartate (NAA)]; for a more detailed review on MRS-rTMS studies, refer to Cuypers and Marsman (2021).

### Healthy Participants

Excitatory rTMS modulates neurotransmitter levels in healthy humans. Iwabuchi et al. (2017) showed a decrease in GABA/Glx ratio in the targeted region (left DLPFC) and the anterior cingulate cortex after iTBS delivery. An early HF-rTMS recorded a decrease in Glx in the stimulated left DLPFC and an increase in the contralateral region (Michael et al., 2003). Interestingly, Glx changes in the left DLPFC negatively correlated with baseline levels, suggesting that baseline Glx might predict rTMS induced Glx changes.

Studies in healthy subjects have reported a relation between brain connectivity and changes in metabolite levels following excitatory rTMS. Iwabuchi et al. (2017) showed that the GABA/Glx ratio decreased in the right DLPFC and was associated with decreased connectivity between the DLPFC and right anterior insula in response to iTBS over the left DLPFC. Another study reported a relation between an increase in GABA/tCr in the posterior cingulate and its connectivity and the inferior parietal lobe (targeted area) after iTBS delivery (Vidal-Piñeiro et al., 2015). These results illustrate the propagation of rTMS effects from the proximal, stimulated area to distal anatomically and functionally connected regions and that GABA and Glx measurements have a significant relationship with the large-scale network changes resulting from rTMS delivery.

Stagg et al. (2009) reported an increase in GABA/NAA ratio in the stimulated motor cortex following a single session of cTBS but could not detect any changes in Glx, possibly due to limited measurement sensitivity. A later cTBS study also reported an increase in GABA concentration in the primary visual cortex, accompanied by a decreased cortical excitability (Allen et al., 2014). Similarly, LF-rTMS also led to increased GABA in the stimulated left motor cortex, accompanied by a GABA decrease in the contralateral M1 (Gröhn et al., 2019), observable after 20 min post-stimulation. These results suggest that LF-rTMS and cTBS stimulation might activate GABAergic interneurons, which in turn modulate excitability in the cortex. Table 2 summarizes MRS findings from healthy participants.

**TABLE 2:** MRS imaging of rTMS effects in healthy controls.

		TMS			MRS		
Author	Subjects	Hz, intensity	Sessions (pulses/session)	Target region	Field strength, Sequence	Voxel location	Results
Michael et al., 2003	*N* = 12	20 Hz, 80% RMT	5 (800)	L DLPC	1.5T, STEAM	L DLPFC R DLPFC L ACC	L DLPFC: ↓ Glx post 1 rTMS session. R DLPFC and L ACC: ↑ Glx pots 5 rTMS sessions.
Stagg et al., 2009	*N* = 16	50 Hz (cTBS) 80% AMT	1 (600)	L M1 Vertex of cranium (sham)	3T, MEGA-PRESS	L M1	↑ GABA/NAA compared to control. NS change Glx/NAA
Allen et al., 2014	*N* = 18	50 Hz (cTBS) 80% RMT	2 (600)	V1	3T, MEGA-PRESS	V1	↑ GABA compared to control
Vidal-Piñeiro et al., 2015	*N* = 36	50 Hz (iTBS or cTBS) session 80% AMT and Sham	1 (600)	L IPL	3T, MEGA-PRESS	L IPL PCC	PCC ↑ GABA/Cr post iTBS compared to sham. PCC GABA increase post iTBS positively related to IPL-to-PCC connectivity
Iwabuchi et al., 2017	*N* = 28	50 Hz (iTBS) at 80% AMT and sham	1 (600)	L DLPFC	3T, PRESS	L DLPFC ACC	L DLPFC and ACC: ↓ GABA/Glx compared to sham
Gröhn et al., 2019	*N* = 7	1 Hz, 90% RMT	1 (1200)	L M1	Laser	L M1 R M1	L M1: ↑ GABA and tCr and ↓ Asp R M1: decrease GABA and Asp

*↓, decrease; ↑, increase; ACC, anterior cingulate cortex; AMT, active motor threshold; DLPFC, dorsolateral prefrontal cortex; Glx, glutamate + glutamine; IPL, inferior parietal lobe; L, left; M1, motor cortex; PCC, posterior cingulate cortex; R, right; RMT, resting motor threshold; V1, visual cortex.*

### Clinical Populations

#### Mood Disorders

In participants with mood disorders, HF-rTMS has been shown to increase GABA concentration in the target area after a clinical course of stimulation over the left DLPFC (Levitt et al., 2019). Similarly, Erbay et al. (2019) reported an increase in Gln/Cr ratio on the left DLPFC after 10 sessions.

Several MRS studies in major depressive disorder participants have reported a relation between metabolite concentration modulation and rTMS therapeutic effects. In work by Croarkin et al. (2016), authors found an increased Gln/Glu ratio following 10 sessions of HF-rTMS over the left DLPFC. These changes in metabolite concentration were accompanied by improvement of depressive symptoms. GABA modulation has also been associated with rTMS therapeutic effects in mood disorders. Increased GABA was reported to be greater in responders (i.e., participants that showed symptom improvement) than in non-responders, even when the change in metabolite concentration was not significant in the whole sample (Dubin et al., 2016; Baeken et al., 2017b). Similarly, in Levitt’s work (Levitt et al., 2019), authors reported a greater increase in GABA concentration in responders than non-responders.

One study also found a relation between baseline metabolite concentration and rTMS therapeutic effects: the authors evaluated the therapeutic effects of 10 sessions of HF-rTMS in patients with mood disorders and found that those who exhibited symptomatic improvement had significantly lower baseline glutamate levels in the left DLPFC, which increased after stimulation (Luborzewski et al., 2007).

#### Other Clinical Populations

Single studies have investigated the modulatory effects of rTMS over GABA, Glx, and glutamate levels in diseases other than mood disorders. In a chronic pain study, LF-rTMS over the right secondary somatosensory increased glutamate at the target and contralateral area (Fregni et al., 2011). Changes in metabolites were accompanied and associated with therapeutic effects. In amyotrophic lateral sclerosis, a study found no significant changes in GABA after bilateral cTBS (Di Lazzaro et al., 2017). However, the authors found a relationship between GABA concentration increase and milder disease progression.

In patients with upper dystonia, delivery of HF-rTMS induced a local decrease in both GABA and Glx, while no changes were observed in distal regions (Marjańska et al., 2013). Importantly, in this study, the authors showed that rTMS modulatory effects of metabolites might differ between patient populations and healthy controls. Their study showed that, opposite to what was found in dystonia patients, HF-rTMS delivery in healthy controls tends to increase GABA in the target region, suggesting a distinct response to rTMS depending on the participant population.

Taken together, these studies illustrate the capacity of rTMS to modulate GABAergic and glutamatergic activity in various populations. Studies of inhibitory rTMS protocols report a consistent increase of GABA concentration in the stimulated region and suggest possible effect of contralateral GABA, while excitatory forms of rTMS report both modulations of GABA and Glx in the targeted region. Furthermore, these studies illustrate the relation between local and distal effects as shown by the correlation between local metabolite concentration changes and distal brain activity (Iwabuchi et al., 2017) and connectivity (Vidal-Piñeiro et al., 2015).

These studies, while plagued by low power, the poor homogeneity of the samples, and the MRS specific technical limitations (voxel size, for example), nevertheless show the profound effects that localized rTMS exerts on the major neurotransmitters of the brain, GABA and glutamate, both acutely and in the long term. They also provide an insight into the therapeutic mechanism of rTMS, as there seems to be a relation between therapeutic outcomes and metabolite concentration modulation. Table 3 summarizes MRS findings from clinical populations.

**TABLE 3:** MRS imaging of rTMS effects in patient population.

		rTMS			MRS		
Author	Subjects (N)	Hz, intensity	Sessions, pulses	Target region	Field strength, Sequence	Voxel location	Results
Fregni et al., 2011	*N* = 17, chronic pain	1 Hz, 70% MSO	10 sessions, 1600 pulses/session	R S2	3T, PRESSS	L S2 R S2	L and R S2: ↑ Glu
Marjańska et al., 2013	*N* = 29, dystonia	5 Hz, 90% RMT	Single session, 1600 pulses	Dominant M1	3T, MEGA-PRESS	Bilateral M1	Dominant M1: ↓ Glx and ↓ GABA
Di Lazzaro et al., 2017	*N* = 3, ALS	cTBS, 80% AMT	120 sessions, 600 pulses/session	Bilateral M1	3T, PRESS and MEGA-PRESS	L M1 WM	No significant changes
Luborzewski et al., 2007	*N* = 17, MD	20 Hz, 100% RMT	10 sessions, 2000 pulses/session	L DLPFC	3T, PRESS	L DLPFC ACC	L DLPFC: lower baseline Glu in responders. ↑ Glu post-rTMS in responders ACC: non-significant change Glu
Croarkin et al., 2016	*N* = 10, MD	10 Hz, 120% RMT	30 sessions, 3000 pulses/session	L DLPFC	3T, PRESS and 2DJ-PRESS	L DLPFC ACC	L DLPFC and ACC: ↑ Gln/Glu 6 months post-rTMS compared to baseline and post-rTMS
Dubin et al., 2016	*N* = 23, MD	10 Hz, range of 80–120% RMT	25 sessions, 3000 pulses/session	L DLPFC	3T, MEGA-PRESS	mPFC	↑ GABA^a^ post-rTMS. Greater GABA^a^ increase in responders
Baeken et al., 2017b	*N* = 36, MD	20 Hz, 110% RMT	20 sessions, 1560 pulses/session	L DLPFC	3T, PRESS	L DLPFC R DLPFC L ACC	L DLPFC: Positive correlation between GABA ↑ and clinical improvement.
Erbay et al., 2019	*N* = 18, MD	10 Hz, –	20 sessions, 2 sessions per day, 3000 pulses/session	L DLPFC	3T, PRESS	L DLPFC	No significant ↑ Glu/Cr
Levitt et al., 2019	*N* = 26, MD	10 Hz, 80–120% RMT	30 sessions, 3000 pulses/session	L DLPFC	3T, MEGA-PRESS	L DLPFC	↑ GABA post-rTMS, greater GABA increase post-rTMS in responders.

*↓, decrease; ↑, increase; ACC, anterior cingulate cortex; ALS, amyotrophic lateral sclerosis; DLPFC, dorsolateral prefrontal cortex; Glx, glutamate + glutamine; IPL, inferior parietal lobe; L, left; M1, motor cortex; MD, mood disorder; mPCC, medial posterior cingulate cortex; R, right; RMT, resting motor threshold; S2, secondary somatosensory; V1, visual cortex.*

*^a^Expresses GABA ratio of peak areas relative to the area of the unsuppressed voxel water signal.*

## Positron Emission Tomography Evaluations of Repetitive Transcranial Magnetic Stimulation Effects in Cerebral Blood Flow and Glucose Metabolism

### Healthy Participants

Early PET studies in healthy subjects showed that both HF- (Paus et al., 1997) and LF-rTMS (Fox et al., 1997) increased rCBF. Similar results were replicated in later studies (Paus et al., 2000; Siebner et al., 2000, 2001b; Speer et al., 2003b; Takano et al., 2004; Knoch et al., 2006).

Early work also suggested the rTMS effect over rCBF to be dose, frequency, and intensity-dependent. More specifically, it was shown that a more significant number of pulses (Paus et al., 1997, 1998), a higher rTMS frequency (Siebner et al., 2001b; Knoch et al., 2006), and a more vigorous stimulation intensity (Speer et al., 2003a,b) induced greater rCBF change. In addition, work from Knoch et al. (2006) suggested rCBF response to rTMS might also vary depending on the stimulated hemisphere. They showed that rTMS over the prefrontal cortex yields activity modulation in different distal regions based on the hemisphere stimulated.

Low-frequency-rTMS modulation of metabolism in the targeted area has been reported to be heterogeneous, with studies reporting both increase (Siebner et al., 1998, 1999, 2001b; Cho et al., 2012; Lee et al., 2013) and decrease (Cho et al., 2012; Lee et al., 2013) after LF-rTMS. HF-rTMS in healthy participants has been only shown to increase local glucose metabolism (Siebner et al., 2000).

Changes in rCBF and glucose metabolism have been mainly localized within the stimulated networks (Paus et al., 1998; Siebner et al., 2001a; Speer et al., 2003b; Takano et al., 2004). Delivery over the motor cortex both increases and decreases activity in the contralateral motor cortex, premotor areas, ACC, cerebellum, and striatum (Paus et al., 1998; Siebner et al., 1998, 2001a; Speer et al., 2003b). Stimulation in the prefrontal cortex modulated rCBF in cognitive regions (Paus et al., 2000; Kimbrell et al., 2002; Speer et al., 2003a; Knoch et al., 2006). Glucose metabolism studies also showed that LF-rTMS delivery decreases activity in the cerebellum and induces a widespread activity modulation in the prefrontal, parietal, and temporal cortex (Cho et al., 2012).

### Clinical Populations

#### Mood Disorders

In an early study, Speer et al. (2000) compared the effect of HF and LF when delivered over the left prefrontal cortex. They found that 10 daily sessions of HF and LF rTMS have opposite effects in local and distal activity modulation in patients with a depressive disorder. In line with the conventionally excitatory and inhibitory convention of HF and LF rTMS, authors found a local and distal increase in rCBF after 20 Hz rTMS while they reported that 1 Hz rTMS induced a decrease in activity. Interestingly, the authors found that patients who showed symptomatic improvement after delivery of one rTMS frequency worsened after the other, suggesting that different mood disorder patients may benefit from different rTMS paradigms.

Repetitive transcranial magnetic stimulation delivery modulates metabolic activity in regions from the cerebellum and the limbic and paralimbic systems (Baeken et al., 2009, 2015; Li et al., 2010). These studies also reported a relation between rTMS induced symptomatic improvement and metabolism modulation. Specifically, Baeken and colleagues demonstrated symptom improvement after HF-rTMS (Baeken et al., 2009), and accelerated HF-rTMS (Baeken et al., 2015) was accompanied by modulation in glucose metabolism of the anterior cingulate, which was not observed in non-responders. These results were further confirmed in other studies that showed HF-rTMS (Li et al., 2010; Takahashi et al., 2013) and iTBS (Li et al., 2018a) induced decrease in glucose metabolism and rCBF in patients that presented symptomatic improvement. The authors also reported a significant correlation between the degree of symptom improvement and the decrease in cingulate cortex metabolism (Baeken et al., 2015).

In line with these results, rTMS antidepressant effects appear predictable based on anterior cingulate metabolism pre-stimulation. Studies have consistently reported higher metabolism in responders to HF-rTMS (Baeken et al., 2009, 2015; Li et al., 2010). Two main observations can be drawn from these findings. One, that rTMS antidepressant effects might be mediated by decreasing the anterior cingulate activity, a region that is metabolically hyperactive during depressive episodes in treatment-resistant depression (Drevets et al., 2008; Mayberg, 2009). And second, that anterior cingulate activity might be a helpful biomarker for early detection of rTMS responders.

These results from PET rCBF and glucose metabolism studies have been replicated using arterial spin labeling, an MRI technique that permits assessments of CBF. Specifically, in a study of major depressive patients, Baeken et al. (2019) reported that decrease in depression severity scores was related to decrease in cerebral perfusion in the frontopolar cortex, a region receiving direct synaptic projections from the DLPFC.

#### Other Clinical Populations

To date, only a few PET studies have evaluated rTMS modulation of metabolic activity in movement disorders. Brusa et al. (2012) reported changes in metabolism following bilateral cerebellar stimulation in patients with levodopa-induced dyskinesia. They found that improvement in motor symptoms accompanied a decrease in metabolism in the cerebellar cortex and deep cerebellar nuclei. These results suggest rTMS therapeutic effects might be facilitated by modulation of these pathways.

Horimoto et al. (2021) showed that concurrent HF and LF rTMS delivery combined with physical therapy in post-stroke hemiplegic patients decreases metabolism asymmetry between the lesional and contralesional areas. The authors also reported that improvement in motor function was associated with changes in metabolism.

Studies in other patient populations have demonstrated metabolism modulation and concomitant symptomatic improvement after rTMS. In subjects with obsessive-compulsive disorder, glucose metabolism of the bilateral orbitofrontal cortex decreases after LF-rTMS, along with a reduction in clinical symptoms (Nauczyciel et al., 2014). In fibromyalgia subjects, increased metabolism in the medial temporal lobe after HF-rTMS over the left M1 was accompanied by an improvement in quality-of-life assessment (Parkitny et al., 2014).

In idiopathic tinnitus, Mennemeier et al. (2011) found local decrease and distal modulation of metabolism accompanied by a significant decrease in tinnitus perception following rTMS. In contrast, Kan et al. (2019) showed that LF-rTMS over the left temporoparietal cortex did not alleviate symptoms, although metabolic activity was increased in the stimulated and remote areas.

Taken together, glucose metabolism and rCBF studies showed rTMS activity modulation to be localized within the stimulated systems, reaching deep brain regions. In patient populations, the changes in activity have seemed to not only be accompanied by symptomatic improvement (Baeken et al., 2009, 2015; Li et al., 2010; Brusa et al., 2012; Nauczyciel et al., 2014) but also correlated to the degree of improvement (Baeken et al., 2015; Horimoto et al., 2021). In patients with mood disorders, anterior cingulate activity is a predictor for rTMS responsiveness (Li et al., 2010, 2018a; Takahashi et al., 2013). Similarly, downregulation of activity in this region modulates rTMS antidepressant effects (Baeken et al., 2015).

## Functional Magnetic Resonance Imaging Evaluations of Repetitive Transcranial Magnetic Stimulation Effects in Resting-State Connectivity

### Healthy Participants

Evaluation of rsFC has been used to reveal the neural mechanisms underlying rTMS effects. In healthy controls, studies have evaluated the effects of HF, LF, and inhibitory and excitatory TBS and QPS delivered mostly over the prefrontal and motor cortices.

The effects of rTMS in rsFC are dependent on the number of stimuli delivered. Nettekoven et al. (2014) found iTBS delivery over the left motor cortex increased connectivity between the stimulated area and the ipsilateral dorsolateral premotor cortex. The increase in connectivity was higher after 1800 pulses compared to changes after 600 and 1200 pulses. An increase in connectivity was also observed between the motor cortex and bilateral areas of the somatosensory and superior parietal cortex, but changes were not dose-dependent, suggesting that the dose-dependent effect might be exclusive to some areas within the stimulated network.

Stimulation of the DLPFC induces a consistent modulation of rsFC across subjects in the anterior cingulate. In a large study carried on 60 healthy participants, Tik et al. (2017) recorded an increase in rsFC in the anterior cingulate cortex after HF-rTMS. These results were supported by a study from a different group, where similar results were observed (Xue et al., 2017).

Bilateral modulation of rsFC has also been reported after QPS delivery to the motor cortex (Watanabe et al., 2014). Excitatory QPS (Interstimulus intervals of 5 ms) decreased rsFC between the stimulated left motor cortex and the contralateral motor cortex, while inhibitory QPS (interstimulus intervals of 50 ms) increased it, illustrating the bidirectional effects of rTMS in rsFC.

Other studies have also reported differences in rsFC response to inhibitory and excitatory rTMS over the motor cortex. Eldaief et al. (2011) found that HF rTMS decreased functional connectivity within nodes of the default mode network when delivered over the posterior inferior parietal lobule, while LF increased connectivity between the targeted area and regions outside the network. A different study reported similar effects after delivery of HF- and LF-rTMS over the medial frontopolar cortex, where authors reported an increase (after HF-rTMS) and decreased (after LF-rTMS) in connectivity between the target area and the amygdala (Riedel et al., 2019). Adding to these findings, Zhang et al. (2020) reported changes in connectivity observed after HF to be more widespread, while LF-rTMS modulated connectivity mostly found in regions within the stimulated hemisphere. This suggests that HF and LF rTMS not only can induce different directional effects but also distinct topological changes.

Similarly, modulation of the motor network with LF rTMS and cTBS, 2 inhibitory forms of TMS stimulation differing by their frequency (1 Hz vs. 50 Hz), has been shown to induce distinct directional effects. Stimulation of the supplementary motor area using LFr-rTMS increased rsFC within the motor network, while cTBS decreased it. Nevertheless, these two stimulation paradigms had similar topographical and temporal effects, with effects being observed after 6 to 10 min post-stimulation (Ji et al., 2017). Similarly, cTBS delivery over the left somatosensory cortex decreased connectivity within the motor network (Mastropasqua et al., 2014; Valchev et al., 2015). Contrary to cTBS studies, HF studies have failed to induce changes in rsFC after stimulation of the supplementary motor area (Hendrikse et al., 2020).

Finally, a cTBS study by Mancini et al. (2017) suggested that recorded changes in functional connectivity might depend on the time they are evaluated. They measured rsFC at 5 and 15 min post stimulation and reported a decreased functional connectivity of the temporal lobe at the 5 min and a change in precuneus connectivity at 15 min.

### Clinical Populations

#### Mood Disorders

Several studies in patients with mood disorders have reported modulation of connectivity in the anterior cingulate after HF-rTMS delivery over the DLPFC (Baeken et al., 2014, 2017a; Salomons et al., 2014; Taylor et al., 2018; Weigand et al., 2018; Ge et al., 2020). Modulation of connectivity between the subgenual cingulate and the orbitofrontal cortex (Baeken et al., 2017a; Taylor et al., 2018), the inferior parietal cortex (Taylor et al., 2018), the occipital cortex (Ge et al., 2020), and the caudate (Salomons et al., 2014) was shown to be associated with symptomatic improvement. Similarly, an iTBS study reported an increase in connectivity between the target area (dorsomedial PFC) and regions from the default mode network and the salience network. This increase in connectivity was predictive of symptom improvement (Persson et al., 2020).

Pre-rTMS connectivity between the anterior cingulate and areas of the prefrontal (Baeken et al., 2014, 2017a; Salomons et al., 2014; Weigand et al., 2018; Ge et al., 2020) and parietal cortices (Ge et al., 2020) have been shown to predict therapeutic response, as higher baseline connectivity was observed in treatment responders. Similarly, in a study of comorbid mood disorders and post-traumatic stress disorder, Philip et al. (2018) found that HF rTMS over the left DLPFC decreased connectivity in the subgenual cingulate, which was increased at baseline. These results are in line with results from Liston et al. (2014) reporting that depressed patients, in comparison with healthy controls, exhibited increased functional connectivity between the subgenual cingulate and nodes of the default mode network such as the ventromedial prefrontal cortex, pregenual anterior cingulate cortex, thalamus, and precuneus. In addition, lower baseline cortico-thalamic, cortico-striatal, and cortico-limbic connectivity have also been reported as predictors of treatment response (Salomons et al., 2014).

#### Movement Disorders

Evaluation of rTMS effects in patients with motor impairment reported a relation between rsFC modulation and therapeutic improvement. In patients with essential tremors, symptomatic improvement after LF-rTMS was accompanied by an increase in connectivity within the cerebello-thalamo-cortical circuit (Popa et al., 2013). In participants with multiple system atrophy, stimulation of the motor cortex improved motor symptoms and modulated rsFC in the cerebellum and regions from the limbic system and the default mode network (Chou et al., 2015). In stroke patients, motor improvement following iTBS delivery and physiotherapy was linked to changes in interhemispheric connectivity rsFC (Volz et al., 2016).

In Parkinson’s patients, LF rTMS over the superior temporal gyrus increased connectivity between the stimulated area and the parahippocampal gyrus, improving dysarthria symptoms (Brabenec et al., 2019), while HF delivery modulated rsFC but did not induce symptom improvement. Flamez et al. (2021) reported that the effect of rTMS on rsFC might be different in Parkinson’s patients with different symptomatic profiles. They found LF-rTMS over the supplementary motor area increased connectivity between the target area and the putamen in patients with levodopa-induced dyskinesia while it decreased connectivity in participants without dyskinesia.

#### Other Clinical Populations

In patients with obsessive-compulsive disorder, a decrease in rsFC in regions of the cortico-striatal-subthalamic circuit correlated with symptomatic improvement after LF (Mantovani et al., 2021) and bilateral HF (Dunlop et al., 2016). In these studies, responders to rTMS exhibited higher pre-rTMS connectivity between the stimulated area and areas from the cortico-striatal-subthalamic circuit (Dunlop et al., 2016; Mantovani et al., 2021). The authors also reported that responsiveness to rTMS might depend on disease severity, as patients with greater impairment showed no improvement following rTMS and showed the strongest baseline connectivity within the cortico-striatal-thalamo-cortical circuit (Mantovani et al., 2021).

In stroke patients, improved cognitive impairment after HF-rTMS correlated to increased functional connectivity of the right medial prefrontal cortex and right ventral anterior cingulate (Yin et al., 2020). In addition, iTBS induced spatial neglect improvement has been linked to greater rsFC modulation within regions of the attention network (Cao et al., 2016). Similarly, an increase in rsFC between the DLPFC and the inferior parietal lobule after iTBS accompanied craving reduction in patients with methamphetamine cravings (Su et al., 2020).

Yet, some studies have failed to report changes in rsFC after rTMS. In a tinnitus study, Roland et al. (2016) found no changes in functional connectivity nor symptom improvement immediately after 2–4 weeks of LF rTMS over the left temporoparietal junction. In patients with disorders of consciousness, Liu et al. (2018) did not find modulation in connectivity after five daily sessions of stimulation nor changes in coma recovery scale scores. In an alcohol dependence study, Perini et al. (2020) also found no changes in symptoms but reported differences in rsFC changes between the sham and HF-rTMS cohort, suggesting that in some instances, modulation of rsFC is not enough to elicit therapeutic effects (Perini et al., 2020).

Overall, rsFC-rTMS studies illustrate the complex network-wise effect of this type of stimulation. In some patient populations, modulation of rsFC is related to symptomatic alleviation and serve as a predictor for treatment responsiveness. In these studies, rsFC changes do not always follow the frequency-dependent convention of inhibitory and excitatory rTMS (Ji et al., 2017), and high and low frequency- rTMS elicit distinct topological effects in rsFC (Zhang et al., 2020). These studies also show that rsFC modulation differs between healthy participants and patient populations (Liston et al., 2014) and even between the same patient population at different disease stages (Flamez et al., 2021).

## Diffusion Tensor Imaging Evaluation of Structural Integrity

Diffusion tensor imaging studies of rTMS effects have been mainly carried in mood disorders and stroke patients. In these studies, authors have reported rTMS induced changes in FA, MD, path length, and cluster coefficient.

### Mood Disorders

Diffusion tensor imaging evaluations have reported FA and MD values modulation. In a study by Kozel et al. (2011), authors found 4–6 weeks of 10 Hz stimulation over the left prefrontal cortex increased FA prefrontal white matter. Similarly, white matter FA in the right superior frontal gyri has been reported to significantly increase after HF-rTMS delivery over the left DLPFC (Tateishi et al., 2019).

Similar to results from functional connectivity studies, a DTI study showed pre-rTMS structural changes in white matter tracks related to therapeutic improvement. A study by Fu et al. (2021) showed baseline FA in fiber tracts from the DLPFC to the insula correlated with symptom improvement in mood disorders patients. The authors found the same correlation when comparing pre-rTMS functional connectivity of the DLPFC and the insula and therapeutic effects. These results illustrate the close relation between fMRI and DTI derived measurements, and more importantly, the similarity between functional and structural connectivity changes induced by rTMS delivery. In a different study, baseline DTI evaluation found reduced white matter FA in the left middle frontal gyrus compared to healthy controls, suggesting abnormalities of white matter in this region might be part of mood disorders pathology (Peng et al., 2012). Authors also reported that 4 weeks of HF-rTMS over the left DLPFC increased FA and induced therapeutic effects; these changes in FA were correlated with therapeutic improvement. Taken together, these results illustrate rTMS capacity to modulate structural changes in regions related to the mood disorders’ pathophysiology and suggest such modulation might facilitate rTMS therapeutic effects.

### Stroke

High-frequency-rTMS has been shown to enhance FA values as soon as 4 days after stroke onset. In a recent clinical trial, the treatment group (those with subcortical stroke) received 10 days of 5 Hz rTMS over the ipsilesional motor cortex. Following the 10 days of rTMS, the treatment group showed increased FA in the contralesional corticospinal tract, the pontine crossing tract, the middle cerebellar peduncle, the contralesional superior cerebellar peduncle, the contralesional medial lemniscus, and the ipsilesional inferior cerebellar peduncle compared to the sham group (Li et al., 2018b). Measures of MD in the superior longitudinal fasciculus and the uncinate fasciculus can be enhanced following a combination of acupuncture therapy and LF-rTMS over the contralesional motor cortex (Zhao et al., 2018). Another study showed that HF- rTMS (10 Hz) to ipsilesional motor cortex enhanced FA values of motor-related gray matter, which correlated with decreased motor impairment. These increases in FA in motor-related white matter were greater than those in a conventional physical therapy group that did not receive HF-rTMS (Guo et al., 2016). Changes in FA values along with symptom improvement suggest rTMS induced recovery of the motor-related system and white matter connections to a greater extent than usual therapy alone.

Yamada et al. (2018) showed that 15 daily LF-rTMS and occupational therapy improved motor function and observed an increase of generalized FA in the white matter of lesioned motor cortex. Similarly, Yang et al. (2015) reported changes in FA following chronic rTMS delivery. Specifically, the authors compared the therapeutic and structural effects of different rTMS frequencies (1 Hz, 10 Hz, and cTBS) in stroke patients with unilateral spatial effect: subjects who received cTBS delivery showed the greatest therapeutic improvement which was accompanied by an increase in FA and MD in the white matter of frontal fasciculus and the capsula interna and externa. Therapeutic effects were also observed after 1 and 10 Hz stimulation, but no changes in FA and MD were reported.

In individuals with aphasia after stroke, five sessions of iTBS applied to the affected hemisphere on separate days showed increases in FA values in several regions of the brain previously shown to have increased fMRI activation during language activation after iTBS (Allendorfer et al., 2012). Specifically, FA was enhanced in inferior and superior frontal gyri, anterior corpus callosum, the right midbrain, and bilaterally near temporal, parietal and posterior cingulate regions in those with aphasia due to stroke.

Diffusion tensor imaging analyzed using graph theory analysis showed that 12 sessions of LF-rTMS over the motor cortex combined with occupational therapy increased path length in the stimulated network in stroke patients (Ueda et al., 2019). These findings suggest rTMS delivery led to more multifaceted neural circuits and, as mentioned by the authors, these new connections might be a compensatory mechanism to cover for the impaired motor paths due to stroke. Graph-theoretical analysis was also used to evaluate DTI measurements before and after rTMS delivery over the left DLPFC in children with cerebral palsy. Zhang et al. (2021) reported an increase in the clustering coefficient of the frontal gyrus and the pallidum, and a decrease of the path length of the right putamen and thalamus, suggesting rTMS stimulation might have improved the efficacy of information exchange through the striatal thalamic projections. These results indicate rTMS delivery modulation of structural connectivity within the corticostriatal pathway and, along with results reported by Ueda et al. (2019), illustrate the complexity of the rTMS effect in brain structure, integrity, and connectivity.

Even though DTI studies of rTMS effects are limited, they show the ability of rTMS to induce changes in white matter microstructure. Several studies have reported modulation of FA values, changes that are thought to represent microstructural changes of white matter tracks (Li et al., 2018b). Through graph theory analysis, studies were also able to measure changes in structural networks and found LF-rTMS might lead the generation of more complex neural circuits, which might ultimately indicate an increase in microstructural connections across the evaluated network. The studies summarized here illustrate the value of DTI-rTMS studies and the ability of rTMS delivery to induce microstructural changes in brain connectivity.

## Caveats

An important limitation of the studies summarized here is the heterogeneity of rTMS parameters. The variability of rTMS protocols and the difference between participant populations limits the comparability between different imaging studies. While there is no one optimal rTMS paradigm, further studies should aim to replicate findings with larger samples and include realistic rTMS sham conditions.

From the studies presented here, it is hard to say whether the effects of rTMS observed in healthy participants can be generalized to disease populations and vice versa. In general, studies in healthy controls have investigated the acute effects of rTMS delivery, where imaging assessments are mostly carried immediately after a single stimulation session due to ethical concerns about unneeded multiple rTMS sessions in healthy participants. Conversely, in clinical populations, rTMS studies mostly evaluate the effects of multiple daily sessions of stimulation. This limits the comparability of and understanding of the neurobiological effects of rTMS between clinical and healthy participants.

## Future Directions

This brief review of the imaging findings after acute or clinical courses of rTMS highlights the important role that neuroimaging tools such as PET, MRS, DTI, and fMRI can play in a better understanding of the physiology, morphology, and neuropharmacology of neural networks, as well as in the preponderant role these non-invasive imaging methodologies can play in designing patient-centered therapies adapted to specific diseases and individual presentations. As these techniques are constantly being developed and improved, the knowledge of how rTMS induces neuroplasticity in the brain grows.

The development of new PET tracers increases our capacity of evaluating the rTMS effect in different neurotransmitters across the brain. For example, the development of tracers targeting the synaptic vesicle glycoprotein (SV2A) (Bretin et al., 2015; Finnema et al., 2016; Nabulsi et al., 2016), a protein critical for synaptic function, could be used to evaluate synaptic changes due to rTMS delivery. SV2A radiotracers such as [11C]UCB-J and [18F]UCB-H are capable of quantifying SV2A density in healthy participants (Finnema et al., 2016; Bahri et al., 2017) and patient populations (Bastin et al., 2020; Holland et al., 2020).

Magnetic resonance imaging techniques such as arterial spin labeling are started to be used in the evaluation of rTMS mechanisms (Orosz et al., 2012; Baeken et al., 2020; Puig et al., 2020; Dalton et al., 2021). Arterial spin labeling has the potential to further advance the current knowledge on stimulation-derived CBF changes.

The development of MR-compatible TMS devices permits the near-simultaneous investigation of TMS actions and brain responses (Navarro De Lara et al., 2015). Similarly, the use of Hybrid PET/MRI has the potential to improve knowledge of and manipulate the neuropharmacology of rTMS mechanisms. On one hand, MRI acquisition can provide information on brain structure, brain metabolites, and neural activity and connectivity. In the other hand, PET can provide complementary information on neurotransmitter release, receptor occupancy, protein expression, glucose and energy metabolism. Thus, PET/MRI is a useful tool for the study of complex effects on the brain, such as the ones elicited by rTMS. The accessibility to such a tool can provide new insights into the induced functional, structural, and neurochemical organization after rTMS.

Positron emission tomography/magnetic resonance imaging studies enable the evaluation of both functional and neurochemical changes. As shown by Sander et al. (2013), simultaneous acquisition of fMRI and PET-raclopride can be used to investigate the relationship between dopamine release and vascular responses following administration of varying doses of dopamine antagonist. Similarly, evaluation of GABA or glutamate (using MRS) and dopamine (using PET) could be carried to better understand the rTMS mechanisms that modulate dopamine release (Schultz et al., 2012; Bunai et al., 2021). Furthermore, the simultaneous acquisition of fMRI and PET- [^11^C]FDG can provide a more complete picture of rTMS induced neuromodulation and changes in functional connectivity (Ionescu et al., 2021). Ultimately, multiple possible combinations for MRI techniques and PET tracers exist, and as the different techniques for PET/MRI analysis and preprocessing are developed, the toolbox for rTMS evaluation increases.

## Conclusion

This review summarizes some of the most relevant findings of rTMS studies carried out using PET and MR techniques. The combination of rTMS and imaging studies provide insight into the neurobiological effects this neurostimulation technique. A better understanding of the neurobiological effects of rTMS will help researchers and clinicians choose more adequate protocols for different patient populations and could help decrease the variability of rTMS effects.

Taken together, results from these studies have illustrated the complex effect of rTMS, as it modulates multiple neurotransmitters and connectivity systems. Specifically, effects in the dopaminergic system are immediate and short-lasting (Strafella et al., 2001; Pogarell et al., 2006), while effects in the serotonergic and opioid system might be longer-lasting (Sibon et al., 2007; Lamusuo et al., 2017). These studies also suggest effects in GABA and glutamate (as measured with MRS), brain activity modulation (as measured with rCBF and glucose metabolism), and functional connectivity might not follow the convention of frequency-dependent inhibition or facilitation. Specifically in the case of functional connectivity, HF- and LF-rTMS yield different topological effects (Zhang et al., 2020).

Regarding rTMS therapeutic effects, most studies reported changes that were often, but not always, accompanied by symptomatic improvement. GABA and Glx concentration, rCBF, and glucose metabolism, as well as functional connectivity have been shown to correlate with therapeutic effects (Baeken et al., 2015; Yin et al., 2020; Horimoto et al., 2021). Finally, studies reported rTMS effects in rsFC are dependent on the population studied, as different responses are observed between healthy controls and patient populations (Liston et al., 2014). RTMS effects may vary depending on the degree of disease progression, as patients with different disease onset have been shown to respond differently to rTMS delivery (Flamez et al., 2021). In conclusion, rTMS neurobiological effects are complex, thus information from multiple imaging methods is required to capitalize on the therapeutic opportunities it presents.

## Author Contributions

LA-S conducted the literature search, prepared the initial draft of the manuscript, created the tables, revised and edited the manuscript. DD supervised all aspects of manuscript preparations and revised and edited the manuscript. JN revised and edited the manuscript. All authors contributed to the article and approved the submitted version.

## Conflict of Interest

The authors declare that the research was conducted in the absence of any commercial or financial relationships that could be construed as a potential conflict of interest.

## Publisher’s Note

All claims expressed in this article are solely those of the authors and do not necessarily represent those of their affiliated organizations, or those of the publisher, the editors and the reviewers. Any product that may be evaluated in this article, or claim that may be made by its manufacturer, is not guaranteed or endorsed by the publisher.
